# Mir-4728 is a Valuable Biomarker for Diagnostic and Prognostic Assessment of HER2-Positive Breast Cancer

**DOI:** 10.3389/fmolb.2022.818493

**Published:** 2022-05-17

**Authors:** Tao Rui, Aizhai Xiang, Jufeng Guo, Ning Tang, Xia Lin, Xin Jin, Jian Liu, Xiaobing Zhang

**Affiliations:** ^1^ Department of Surgery, Affiliated Hangzhou First People’s Hospital, Zhejiang University School of Medicine, Hangzhou, China; ^2^ The Center for Integrated Oncology and Precision Medicine, Affiliated Hangzhou First People’s Hospital, Zhejiang University School of Medicine, Hangzhou, China; ^3^ NHC Key Laboratory of Combined Multi-organ Transplantation, Hangzhou, China

**Keywords:** breast cancer, HER2, mir-4728, early diagnosis, ESR

## Abstract

Breast cancer remains one of the most common malignancies in female cancer patients. The rapid and accurate diagnosis of human epidermal growth factor receptor 2 (HER2) status is indispensable for breast cancer patients. The pre-miR-4728 (mir-4728) is encoded within an intron of the HER2 gene. We showed here that mir-4728 was the most significantly upregulated pre-miRNA in HER2-positive breast cancer patients (fold-change: 4.37), and it could serve as a strong diagnostic factor for the HER2 status in breast cancer patients (*p* < 0.0001). Moreover, mir-4728 was positively correlated with tumor recurrence and appeared to be a critical independent risk factor of tumor recurrence in patients with high tumor burden (HR: 7.558, 95% CI:1.842-31.006, *p* = 0.005). Remarkably, HER2-positive patients with higher mir-4728 expression levels had better drug responses to targeted therapies. Furthermore, estrogen receptor (ESR), the predictive marker for endocrine therapies, was found to be the direct target of miR-4728-3p. Taken together, our results supported the potential role of mir-4728 in the diagnosis of HER2 status and the prognostic assessment of HER2-positive patients in response to targeted therapies.

## Introduction

Although multimodality treatment has contributed to improving breast cancer prognosis, breast cancer remains one of the major causes of cancer mortality in female cancer patients (DeSantis et al., 2019; Siegel et al., 2019). Human epidermal growth factor receptor 2 (HER2)-positive breast cancer is a particular class of breast cancer, which accounts for 25% of all breast cancers and has a poor prognosis. Fortunately, comprehensive targeted therapy can significantly prolong HER2-positive patient survival (Ross and Gray, 2003; Escrivá-de-Romaní et al., 2018). Thus, an accurate and rapid diagnosis of the HER2 status is essential for the timely treatment of breast cancer patients. Currently, the technique of HER2 diagnosis depends on immunohistochemistry (IHC) (Pauletti et al., 2000). The patients whose IHC score is 3+ could be diagnosed with a HER2-positive status, while the patients with an IHC score of 2+ must be further detected with fluorescent *in situ* hybridization (FISH) assay (Carlson et al., 2006), which is costly and not widely available. Only a few qualified institutions could provide such diagnostic services. Therefore, it is necessary to explore a more straightforward method to examine the HER2 status.

The novel mir-4728 (pre-miR-4728) is encoded within an intron of the HER2 (ErbB2) gene (Ch17:39726495-39726561) (Persson et al., 2011). A co-amplification or co-expression might exist between mir-4728 and HER2. Thus, we hypothesized that the expression of mir-4728 could reflect the HER2 status of breast cancer. The *in vitro* functions of mir-4728 in promoting breast cancer malignancy have been reported (Li et al., 2015; Floros et al., 2018). However, the correlation between mir-4728 expression and HER2 status in breast cancer remains to be explored, especially the potential application of mir-4728 in clinical diagnosis.

The status of estrogen receptor (ESR) or progestogen receptor (PGR) also guides the treatment of breast cancer patients in clinical applications. After combining systematic endocrine therapy, ESR and PGR-positive breast cancer patients can receive a favorable prognosis (Hammond et al., 2011; Jorns, 2019). As three key markers for breast cancer, the association between the status of HER2 and ESR or PGR warrants further attention and investigation. In this study, we focused on the potential value of mir-4728 in the diagnosis of HER2 status and prognostic evaluation for breast cancer. The correlation between mir-4728 and ESR or PGR was also established.

## Materials and Methods

### Data Acquisition and Analysis

As described previously (Rui et al., 2020a), the miRNA-sequencing data, mRNA-sequencing, and the matched clinical data of breast cancer samples and non-tumor samples were downloaded from The Cancer Genome Atlas (TCGA) database (https://cancergenome.nih.gov/). The HER2 status of breast cancer patients has been diagnosed in TCGA database. By using the “edgR” R software package, the differentially expressed miRNAs in HER2-positive breast cancer patients were normalized, screened, and compared with HER2-negative patients. Log_2_ fold change > 1 and FDR (false discovery rate) < 0.05 were set as the satisfying criteria. The complete clinicopathological information of the patients was also acquired from TCGA database. Clinical characteristics for the patients from TCGA are shown in Supplementary Table S1.

### Cell Culture

The breast cancer cell lines, MCF-7 and ZR-75-1, were purchased from the Cell Bank of the Chinese Academy of Sciences. The Roswell Park Memorial Institute 1640 Medium (GIBCO, United States) with β-estradiol (1 nM) was for ZR-75-1. The Minimum Essential Medium (GIBCO, United States) was for MEC-7. All media were supplemented with 10% FBS (BioIND, China). The cells were cultured in a humidified incubator with the condition of 5% CO_2_ at 37°C.

### Cell Transfection

For evaluating the targets of miR-4728-3p and miR-4728-5p in breast cancer, miR-4728-3p mimic oligonucleotides, miR-4728-5p mimic oligonucleotides, and their controls (RiboBio, China) were synthesized. The efficient RNA transfection reagent RiboFECT (RiboBio, China) was used to transfect the RNA oligonucleotides (250 nM) according to the manufacturer’s instructions.

### Dual-Luciferase Reporter Assay

As described previously (Rui et al., 2021), the pmirGLO vector (Repobio, China) was designed to assess the inhibitory effect of miRNAs by measuring the luciferase activity. The sequences of wild-type or the mutant-type 3’ untranslated regions (3’UTRs) of the ESR were cloned into the pmirGLO vector. Then, the 293T cells were co-transfected with the constructed vectors and miR-4728-3p mimics or control mimics by using lipofectamine 3000 (Thermo Fisher Scientific, United States). After 48 h, a dual-luciferase activity was detected with the Dual-Luciferase Reporter assay kit (Vazyme, China), according to the manufacturer’s instructions.

### Western Blot Analysis

As described previously (Rui et al., 2020b), the total proteins extracted from the MFC-7 and ZR-75-1 were quantified and boiled. Then, the total proteins were electrophoresed with 10% SDS-PAGE gel (FD341-100, Fudebio, China) and transferred onto equilibrated polyvinylidene-difluoride membranes. The membranes were blocked with 5% BSA for 1 hour at room temperature and incubated with anti-ESRα (A0296, Abclonal, China), anti-PGR (A0321, Abclonal, China), and anti-GAPDH (A19056, Abclonal, China) overnight at 4°C. Before being detected by an enhanced chemiluminescence system (Biotanon, China) with FDbio-FemtoEcl (FD8380, Fudebio, China), the membranes were incubated with the secondary anti-rabbit IgG HRP-linked antibody.

### Statistical Analysis

The quantitative data were expressed as the mean ± standard deviation (SD) or median ± interquartile range (IQR). The Student’s *t*-test or Mann–Whitney test was performed to compare the quantitative variables between the two groups. The optimal cutoff value of mir-4728 for diagnosing the HER2 status of breast cancer was detected with the ROC curve, by calculating the best Youden index, considering both sensitivity and specificity. A one-way ANOVA followed by a post hoc Bonferroni test was used to analyze more than two groups. Categorical measures were compared with the chi-square test or Fisher exact test. The Kaplan–Meier survival curves were assessed with the log-rank test. The independent risk factors of breast cancer recurrence were screened with the multivariate Cox proportional hazards regression analysis. A statistical analysis was performed with the SPSS Software (Version 19.0), and the *p*-value < 0.05 was set as the significance level. “^∗^”, “^∗∗^”, “^∗∗∗^” and “^∗∗∗∗^” indicated *p*-value < 0.05, 0.01, 0.001, and 0.0001, respectively.

## Results

### Mir-4728 Is a Strong Diagnostic Factor for the Human Epidermal Growth Factor Receptor 2 Status of Breast Cancer Patients

**FIGURE 1:**
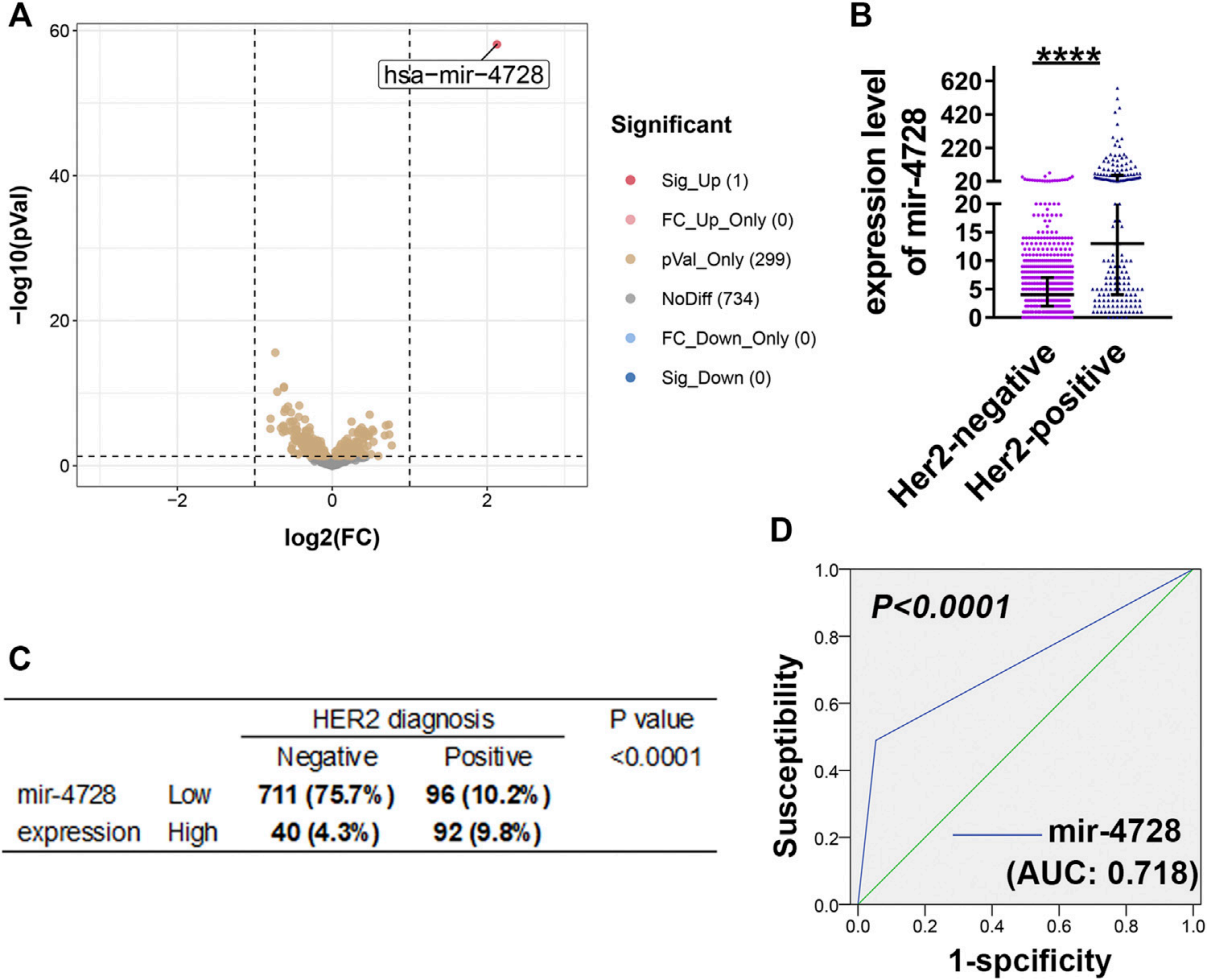
Mir-4728 acts as a key marker in the diagnosis of HER2 status. **(A)** Volcano plot depicts the differentially expressed mirnas between breast cancer patients with HER2-positive status and HER2-negative status. **(B)** Mir-4728 is significantly upregulated in HER-2 positive patients in comparison with HER2-negative patients. **(C)** Correlation analysis between the mir-4728 expression and HER-2 status diagnosis in breast cancer patients. **(D)** The area under the receiver operating characteristic (AUROC) shows that mir-4728 significantly predicts the HER-2 status diagnosis in breast cancer patients.

Based on the miRNA sequencing results and HER expression levels from TCGA, we enrolled the data of 939 breast cancer samples. Then, we divided the patients into HER2-positive and HER2-negative groups according to the HER2 status diagnosis and screened the differentially expressed miRNAs (at the criteria of log_2_ fold-change > 1 and FDR > 0.05). We found that mir-4728 was a unique miRNA with the most significant and highest expression in HER2-positive breast cancer patients (Figure 1A; Supplementary Material S1), with a fold-change up to 4.37 (Figure 1B). The results showed that the HER2-positive patients expressed significantly higher levels of mir-4728, which indicated the potential links between the mir-4728 expression level and the HER2-positive status. Thus, using the chi-square test, we confirmed that the expression of mir-4728 was significantly correlated with HER2 status (Figure 1C). Low mir-4728 levels were particularly associated with a HER2-negative status. The ROC curve further showed that mir-4728 expression levels predicted the HER2 status in breast cancer patients. The area under the curve (AUC) was 0.718 (*p* < 0.0001) (Figure 1D). The values of specificity and sensitivity were 94.9 and 66.2%, respectively. The positive predictive value and negative predictive value were 82.5 and 88.7%, respectively. The results suggested that mir-4728 might be a valuable marker for the prediction of HER2 status in breast cancer patients.

### Mir-4728 Predicts the Early Recurrence of Breast Cancer and Guides the Therapy for Breast Cancer

**FIGURE 2:**
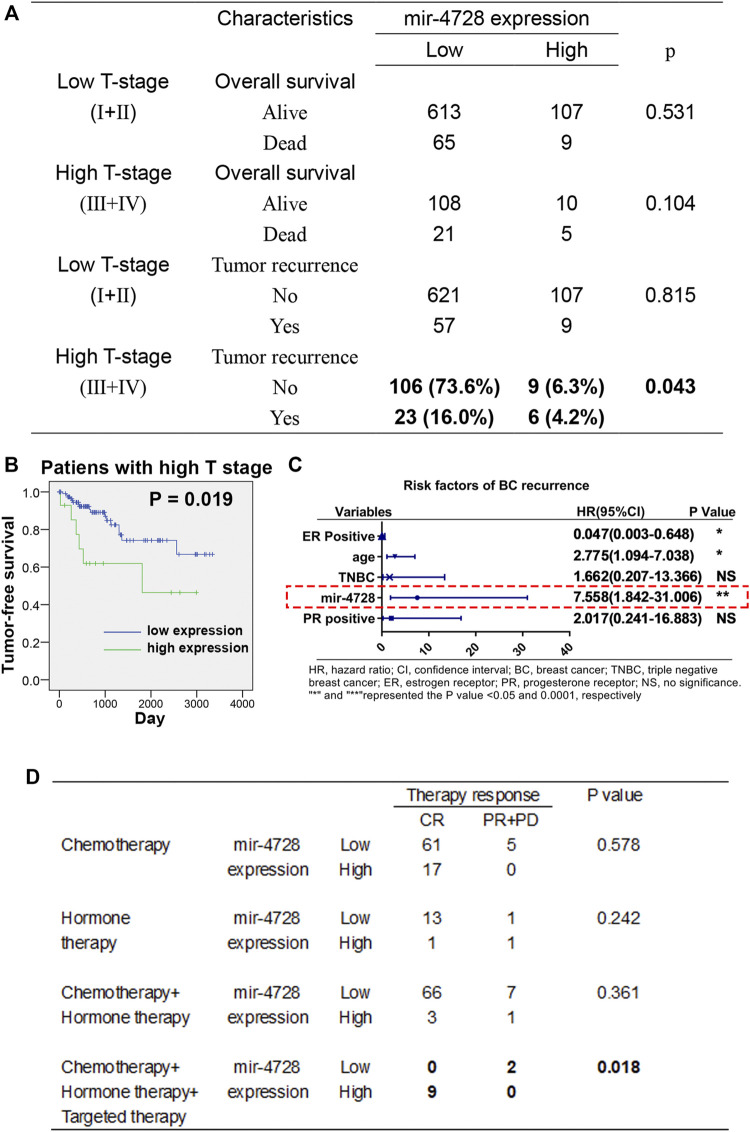
Potential clinical implication of mir-4728 in breast cancer patients. **(A)** Correlation analysis between the mir-4728 expression and tumor recurrence in breast cancer patients at the high T and low T stages. **(B)** Log-rank statistics show that high mir-4728 expression predicts early tumor recurrence in the patients with high T stage. **(C)** Independent risk factors of tumor recurrence in patients at high T stage. **(D)** Correlation analysis between the mir-4728 expression and therapy response in the patients with different therapeutic strategies.

We further evaluated the utility of mir-4728 in the prognosis assessment of breast cancer. Using the chi-square test, we found that in breast cancer patients at the high T stage, the mir-4728 expression was positively correlated with disease recurrence (*p* = 0.043) but not with tumor survival (Figure 2A). The Kaplan-Meier analysis with log-rank test also confirmed that higher expression of mir-4728 predicted lower tumor-free survival, only in the high T stage patients (*p* = 0.019) (Figure 2B; Supplementary Figure S1). A multivariate Cox proportional-hazards model showed that mir-4728 was the most important independent risk factor of tumor recurrence in patients at the high T stage (HR: 7.558, 95% CI:1.842-31.006, *p* = 0.005) (Figure 2C). These results suggested that mir-4728 had a significant predictive value in the recurrence of high tumor burden in breast cancer.

Given the intensive correlation between the expression of mir-4728 and HER2 status in breast cancer patients, we speculated a potential role of mir-4728 in guiding drug therapy for the patients. Following the designed therapeutic regimens, we stratified the patients into four treatment groups (chemotherapy; hormone therapy (endocrine therapy); chemotherapy combined with hormone therapy; and hormone therapy combined with targeted therapy). The results indicated that patients with targeted therapy who expressed higher mir-4728 responded better (all nine patients were in complete remission) (*p* = 0.018) (Figure 2D). This indicated that mir-4728 could predict the effect of targeted therapy and guide drug therapy against breast cancer. However, because of the small number of patients enrolled, the results need to be further verified with much more extensive clinical samples.

### Estrogen Receptor But Not Progestogen Receptor is the Direct Target of miR-4728-3p

**FIGURE 3:**
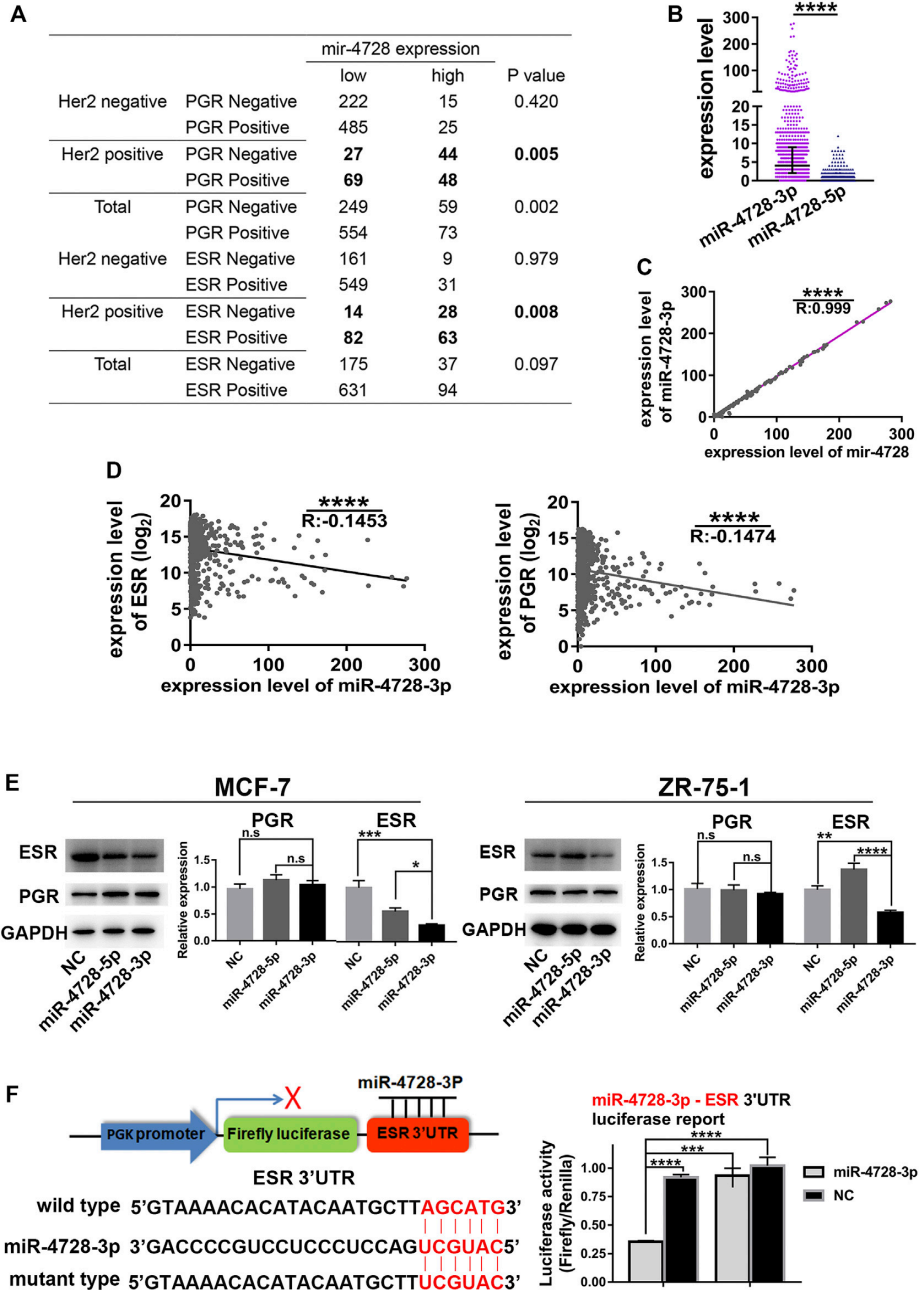
MiR-4728-3p, spliced from mir-4728, directly targets the expression of ESR. **(A)** Correlation analysis between the expression of mir-4728 and ESR or PGR in the patients with different HER2 statuses. **(B)** The expression of miR-4728-3p and miR-4728-5p in breast cancer. **(C)** Linear correlation analysis between the expression of mir-4728 and miR-4728-3p. **(D)** Linear correlation analysis between the expression of miR-4728-3p and ESR (left) or PGR (right). **(E)** Western blot detects the change of ESR and PGR expression under the control of miR-4728-3p, miR-4728-5p, or the controls. **(F)** Dual-Luciferase assay shows the relative luciferase activities (Firefly/Renilla) among the 293T cells co-transfected with miR-4728-3p or controls and wild-type or mutant-type Dual-Luciferase reporters.

Interestingly, data from TCGA indicated that the expression of mir-4728 was negatively associated with ESR-positive and PGR-positive diagnoses, especially in HER2-positive patients (Figure 3A). We suspected that in HER2-positive patients, one of the mature miRNAs, miR-4728-3p or miR-4728-5p, spliced from the high expression of mir-4728, might inhibit the expression of ESR or PGR. First, we must determine the mature functional form of mir-4728. We found that the expression of miR-4728-3p was significantly upregulated in breast cancer than that of miR-4728-5p (fold-change: 30.33) (Figure 3B). A linear regression analysis also showed that there was an extremely significant correlation between miR-4728-3p and mir-4728 (R: 0.999, *p* < 0.0001) (Figure 3C). All the results showed that miR-4728-3p was the mature functional miRNA spliced from mir-4728.

Next, we further attempted to detect whether miR-4728-3p could inhibit the expression of ESR or PGR. The mRNA levels of ESR and PGR from TCGA database were acquired. A linear regression analysis showed that miR-4728-3p was significantly negatively correlated with both ESR and PGR levels, indicating that miR-4728-3p might inhibit the expression of ESR and PGR (Figure 3D). The experiment was also performed to confirm that ESR and PGR expression changes under the control of miR-4728-3p. First, the ESR and PGR-positive breast cancer cell lines MCF-7 and ZR-75-1, were transfected with miR-4728-3p mimics, miR-4728-5p mimics, or their controls, respectively. A Western blot showed that miR-4728-3p significantly inhibited the expression of ESR, which did not occur with the miR-4728-5p and control counterpart (Figure 3E). However, the PGR expression was not markedly changed by miR-4728-3p, indicating that PGR was not the target of miR-4728-3p (Figure 3E). To further confirm that ESR was the direct target of miR-4728-3p in breast cancer, we predicted the potential target sites of 3'UTR of ESR from the miRWalk database (http://mirwalk.umm.uni-heidelberg.de) (Sticht et al., 2018). The wild-type and mutant luciferase reporter vectors of ESR-3'UTR were constructed. Then, the miR-4728-3p mimics or control mimics and wild-type 3'UTR vector or mutant 3'UTR vector were co-transfected into 293T cells, for the Dual-Luciferase Reporter assay. The results showed that the activity of luciferase was significantly inhibited in the miR-4728-3p-wild-type vector group (Figure 3F). Thus, miR-4728-3p, the functional form of mir-4728, could directly target the expression of ESR.

## Discussion

The status of HER2, ESR, PGR, and Ki67 guide the classification of the four molecular subtypes of breast cancer (Perou et al., 2000; Sørlie et al., 2001). Meanwhile, HER2 status basically guides the whole process of breast cancer treatment. Not only does targeted therapy (trastuzumab, pertuzumab, and TDM-1) depend on the diagnosis of HER2 status, but the HER2 status also affects the chemotherapy strategy for breast cancer (Gradishar et al., 2020). Therefore, it is necessary to diagnose the HER-2 status before treating breast cancer. The diagnosis of HER2 status relies on the IHC and FISH analyses. However, it is equivocal by FISH when HER2: CEP17 (centromere) is < 2.0, but HER2 signals are  ≥ 4 but < 6 (Agersborg et al., 2018). In this study, we aim to discover the ideal biomarker for easily and accurately diagnosing the HER2 status of breast cancer. MiRNAs or pre-miRNAs, as one kind of small non-coding RNAs, are the emerging biomarker candidates in multiple cancers (Bjorkman and Taylor, 2019). Persson et al. (2011) showed that mir-4728 is from the intron of the HER2 gene, suggesting that the expression of mi-4728 may reflect the HER2 status. Li et al. (2015) also confirmed that miR-4728-3p is upregulated in HER2-positive breast cancer patients. However, the exact diagnostic value of mir-4728 to HER2 status remains unclear. We showed in this study that mir-4728 was a unique miRNA with the most significant high expression in HER2-positive patients, compared with the HER2-negative patients. The ROC curve indicated that mir-4728 could well predict the HER2 status of breast cancer, with the specificity being as high as 94.7%. Our results strongly suggested that mir-4728 could serve as a biomarker for the diagnosis of HER2 status.

Clinically, compared with the other two molecular subtypes (luminal A and luminal B), patients with a HER2-positive status have poorer survival and a higher rate of distant metastasis (Kennecke et al., 2010; Godoy-Ortiz et al., 2019). In view of the high correlation between mir-4728 and HER2 status, we speculated that mir-4728 could have a predictive role in the prognosis of breast cancer. Our results confirmed that mir-4728 predicted the early recurrence of breast cancer patients with a high tumor burden. Meanwhile, mir-4728 was the independent risk factor for breast cancer recurrence. High tumor burden breast cancer patients who had high levels of mir-4728 had a remarkably high recurrence risk (HR: 7.558, 95% CI: 1.842-31.006). The breakthrough of targeted therapy can provide more therapeutic options for HER2-positive breast cancer patients. Trastuzumab, pertuzumab, and TDM-1 have offered a better prognosis for HER2-positive patients (Loibl and Gianni, 2017). Interestingly, our data suggested that HER2-positive patients with a high mir-4728 expression could respond better to targeted therapy. However, the results based on TCGA database are considered as incomplete and preliminary. Thus, a clinical trial to confirm that mir-4728 could guide the targeted therapy of HER2-positive breast cancer is warranted.

ESR and PGR-positive patients who receive endocrine therapy can have a better prognosis (Cheung et al., 2000; Early Breast Cancer Trialists' Collaborative Group (EBCTCG), 2005). So far, there are few studies on the association between mir-4728 and ESR or PSR status. Our analyses indicated that there were significant negative correlations between mir-4728 and ESR or PGR status. However, when we transfected the miR-4728-3p or miR-4728-5p into breast cancer cell lines, the expression of PGR was not inhibited, suggesting that PGR was not the direct target of mir-4728. The indirect connection and potential regulatory mechanisms between mir-4728 and PGR status may deserve further exploration. Previously, Newie et al. (2014) showed another seed site of ESR 3’UTR that miR-4728-3p binds to. In this study, we confirmed that miR-4728-3p was the functional miRNA spliced from mir-4728. Through Western blotting and dual-luciferase reporter assay, we demonstrated that ESR was the direct target of miR-4728-3p. These results suggest that the poor prognosis of HER-positive status in breast cancer may be partly due to the suppression of ESR by the co-expression of mir-4728.

There have been limitations in this study. The results came from the sequencing data of the TCGA database. Therefore, it should be further verified in clinical samples. Also, this study showed the correlation between PGR and mir-4728 but failed to conclude the inner mechanisms, which deserved attention.

In summary, we showed a novel role of mir-4728 in the diagnosis and prognosis prediction of breast cancer. Mir-4728 represents an excellent biomarker for the prediction of the HER2 expression status in breast cancer patients. Meanwhile, mir-4728 can predict the poor prognosis of high tumor burden breast cancer patients and may evaluate the therapeutic effect of targeted therapy for breast cancer patients. ESR, but not PGR, is confirmed as the direct target of miR-4728-3p. This work shed light on the clinical use of mir-4728, which can become a valuable biomarker for the diagnosis of HER2 status and the assessment of therapeutic effects on breast cancer if given a multi-center prospective clinical study validation.

## Data Availability

The original contributions presented in the study are included in the article/Supplementary Material, further inquiries can be directed to the corresponding author.

## Abbreviations

AUC, area under the curve; ESR, estrogen receptor; HER2, human epidermal growth factor receptor 2; mir-4728, pre-miR-4728; mirna, pre-miRNA; PGR, progestogen receptor; TCGA, The Cancer Genome Atlas; 3’UTR, 3' untranslated region.
