# Supplementary material for: Mir-4728 is a Valuable Biomarker for Diagnostic and Prognostic Assessment of HER2-Positive Breast Cancer
Source: Front Mol Biosci. 2022 May 17;9:818493. doi: 10.3389/fmolb.2022.818493 (PMC9152170; doi:10.3389/fmolb.2022.818493)
Supplement: Supplementary file 2 [file DataSheet1.docx]

**Supplementary Table S1**

We obtained data of 1103 breast cancer patients from the TCGA database. Finally, 939 cases which had complete mir-4728 expression level, HER2 expression level were enrolled in this study.

**Table.S****1: Clinical characteristics for the breast cancer patients from TCGA**

| Factor | Mean ± SD |  | n | % |
| --- | --- | --- | --- | --- |
| Age (years) | 58.74 ± 13.19 |  |  |  |
|  |  | ≥ 65 | 661 | 70.4% |
|  |  | < 65 | 278 | 29.6% |
| Gender |  | Female | 928 | 1.2% |
|  |  | Male | 11 | 98.8% |
| mir-4728 expression |  | Low | 807 | 85.9% |
|  |  | High | 132 | 14.1% |
| T stage |  | Low (stage I+II) | 794 | 84.6% |
|  |  | High (stage III+Ⅳ) | 144 | 15.4% |
| Lymph node metastasis |  | Low (N0+N1) | 753 | 81.3% |
|  |  | High (N2+N3) | 173 | 18.7% |
| AJCC stage |  | Low (stage I+II) | 702 | 75.9% |
|  |  | High (stage III+Ⅳ) | 223 | 24.1% |
| Tumor recurrence |  | Negative | 843 | 89.8% |
|  |  | Positive | 96 | 10.2% |
| Tumor event |  | Survival | 839 | 89.4% |
|  |  | Dead | 100 | 10.6% |
| ER status |  | Negative | 212 | 22.6% |
|  |  | Positive | 725 | 77.4 |
| PR status |  | Negative | 308 | 32.9% |
|  |  | Positive | 627 | 67.1% |
| HER2 status |  | Negative | 751 | 80.0% |
|  |  | Positive | 188 | 20.0% |

Abbreviations: ER: Estrogen Receptor; PR: Progesterone Receptor; HER2: Human Epidermal Growth Factor Receptor 2.

**Supplementary Figure S1:**


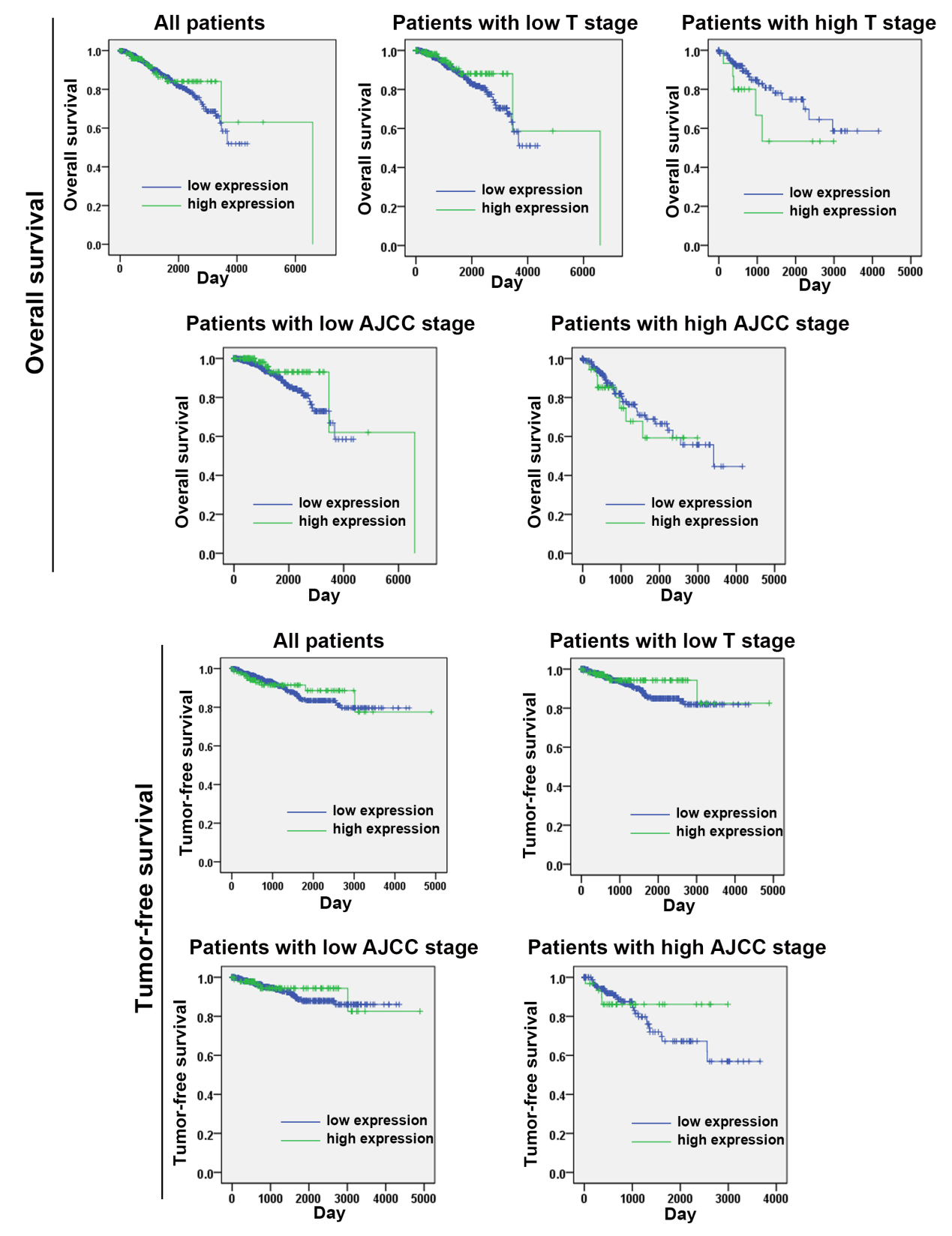


Supplementary Figure S1: The mir-4728 expression levels failed to predict the poor overall survival after stratifying the breast cancer patients into different groups, such as low T stage, high T stage, low AJCC stage, and high AJCC stage. And the mir-4728 could not predict the poor tumor-free survival of breast cancers patients with low T stage, low AJCC stage, and high AJCC stage. (All p value > 0.05).
